# MiR-7e-5p downregulation promotes transformation of low-grade follicular lymphoma to aggressive lymphoma by modulating an immunosuppressive stroma through the upregulation of FasL in M1 macrophages

**DOI:** 10.1186/s13046-020-01747-z

**Published:** 2020-11-09

**Authors:** Xiaoli Lou, Jianhong Fu, Xin Zhao, Xuemei Zhuansun, Chao Rong, Maomin Sun, Hui Niu, Lei Wu, Yongsheng Zhang, Lu An, Lingchuan Guo, Shan Wan, Shouli Wang

**Affiliations:** 1 Department of Pathology, School of Biology & Basic Medical Sciences, Soochow University, Suzhou, 215123 China; 2 Department of Pathology, the Second Affiliated Hospital of Soochow University, Suzhou, 215004 China; 3 Department of Hematology, the First Affiliated Hospital of Soochow University, National Clinical Research Center for Hematologic Diseases, Soochow University, Suzhou, 215006 China; 4 Laboratory Animal Research Center, Soochow University School of Medicine, Suzhou, 215123 China; 5 Department of Pathology, the First Affiliated Hospital of Soochow University, Suzhou, 215006 China; 6 Collaborative Innovation Center of Clinical Immunology between Soochow University and Sihong People’s Hospital, Sihong, 223900 China

**Keywords:** Follicular lymphoma, miR-7e, C-MYC, FasL, Macrophage, PARP

## Abstract

**Background:**

In follicular lymphoma (FL), histologic transformation to high-grade FL and diffuse large B-cell lymphoma (DLBCL) is a critical adverse step in disease progression. Activation of the oncogene c-MYC and tumor microenvironment remodeling account for FL progression. A panel of microRNA (miRNA) was downregulated in transformed FL (tFL).

**Methods:**

Differentially expressed miRNAs were systematically compared in 11 lymph nodes from patients at different stages of disease. Expression of miR-7e-5p was analyzed in 46 B-cell lymphomas, including 30 FL tissues and 16 DLBCL tissues. In FL cells, transcriptional regulation of the oncogene c-MYC on its target miR-7e-5p was revealed by Chromatin Immunoprecipitation (ChIP) assay. Exosome, carrying differentially expressed miR-7e-5p was isolated and visualized by transmission electron microscope and fluorescence tracing. The effect of miR-7e-5p on recipient macrophage was determined by target gene quantification, flow cytometry, and TUNEL method in a cocultured system with miR-7e-5p-mimics or inhibitors treatment. Expression of miR-7e-5p targets, macrophage proportions, and clinical parameters were included for correlation analysis.

**Results:**

We determined that downregulation of miR-7e-5p, driven by c-MYC overexpression, was associated with poorer prognosis in FL patients. The decreased expression of miR-7e-5p in lymphoma cells led to a reduced exosomal transfer to surrounding macrophages. As a result, the target gene of miR-7e-5p, Fas ligand (FasL), was upregulated and activated the caspase signaling, which led to the apoptosis of M1 macrophages in tumor stroma. Finally, in transformed FL tissues, overexpression of FasL and activation of caspase proteins was detected in tumor stromal macrophages. Downregulation of miR-7e-5p was associated with poorer clinical outcomes.

**Conclusion:**

Downregulation of exosomal miR-7e-5p induces stromal M1 macrophage apoptosis, which leads to immunosurveillance and transformation of FL.

**Supplementary Information:**

The online version contains supplementary material available at 10.1186/s13046-020-01747-z.

## Background

Follicular lymphoma (FL), an indolent B-cell lymphoma, can histologically transform into high-grade FL and a highly aggressive malignancy, diffuse large B-cell lymphoma (DLBCL), under certain conditions. The malignant transformation of FL is considered an adverse step for patients and is associated with a much poorer clinical course and outcomes [1]. Transformation has been found to occur in approximately 30% of patients at 10 years, which is associated with a decline in survival to less than 2 years [1]. For this reason, understanding the pathogenesis of FL transformation is helpful for the prediction and treatment of FL patients. Accumulation of genetic alterations has been found to be important for FL transformation. For example, patients with follicular lymphoma always show more aggressive clinical outcomes with rearrangement of the oncogene MYC.

In addition to genetic alterations, the tumor microenvironment is another important aspect that affects FL progression [2]. Decreased subpopulations of CD4+/CD8+ T cells, macrophages and dendritic cells in patients are associated with FL transformation and are predictors of worse survival [2–4]. In a large cohort of 132 FL patients, elevated levels of programmed death ligand-2 (PD-L2) expression and reduced intratumoral immune infiltration were correlated with adverse outcome [5]. A more specific subgrouping of PD-1-positive cells, which further separates them into *T follicular helper* (TFH) cells and exhausted T cells, enables the prediction of acute progressive FL according to the ratio between TFH and exhausted T cells [2]. These population-based clinical data provide reliable evidence that the homeostasis of the immune microenvironment strongly influences the fate of FL.

Recently, exosomes have been found to act as essential mediators in the maintenance and modulation of the tumor microenvironment [6]. One of the active substances transported by exosomes is microRNA (miRNA), which is a single-stranded RNA that fine tunes the expression of target genes at the posttranscriptional level [7]. Being transported by exosomes, tumor-derived miRNAs are delivered to neighboring immune cells and suppress their cytotoxic activity against tumor cells [7]. In addition to tumor parenchyma, miRNAs can also be produced by stromal cells, which in turn affects tumor growth and invasion [8]. In a mouse model, miR-298-5p from activated CD8+ T cells induced the apoptosis of mesenchymal stem cells, which prevented the formation of mesenchymal tumor stroma and tumor cell metastasis [8].

Exosomal miRNAs act as important mediators that maintain the tumor microenvironment and are regarded as potential therapeutic methods for different tumors [7, 8]. In the context of FL, a panel of miRNAs that includes miR-7e, miR-30a, and miR-199a has been found to be associated with disease progression [9, 10]. Specifically, miR-150 negatively regulates *forkhead box transcription factor (FOX)-P1*, which inhibits the progression of lymphoid malignancies, while miR-31 targets *E2F transcription factor 2* (E2F2) and *class II PI3 kinases* (PIK3C2A) and inhibits tumor cell proliferation in FL [9, 10]. Although these studies reveal the tumor suppressive role of miRNAs, little is known about their function in modulating the tumor microenvironment. Given the diverse functions of miRNAs observed in other tumor types, certain miRNAs may contribute to the maintenance of microenvironmental homeostasis, which prevents the progression of FL.

In this study, we screened for miRNA candidates in patient tissues that play important roles in FL transformation. We identified miR-7e-5p, which was downregulated from indolent FL to high-grade FL and DLBCL. By using a coculture system and exosome isolation, we detected the transfer of miR-7e-5p from tumor cells to surrounding M1 macrophages. Repressed expression of miR-7e-5p led to the upregulation of FasL and activation of caspase signaling, which accounted for the apoptosis of M1 macrophages. Clinical data demonstrated the correlation of miR-7e-5p downregulation, upregulation of downstream targets and clinicopathologic features in FL transformation.

## Materials and methods

All antibodies and RNA information were listed in Supplementary Table S1–S2.

### Patient samples

Patient tissue samples used in this study were collected from lymph nodes, which were diagnosed as FL or DLBCL following the World Health Organization (WHO) guidelines [11]. Duodenal-type follicular lymphoma and other extranodular lymphoma were excluded. Eleven frozen neoplastic lymph node tissues, consisted of seven FL cases (grade 1: *n* = 2, grade 2: *n* = 1, grade 3A: n = 1, grade 3B: *n* = 3), and four DLBCL cases were examined for six candidate miRNAs. For miR-7e-5p quantification, frozen tissues from neoplastic lymph nodes (*n* = 46), including different stages of FL cases (*n* = 30) and DLBCL cases (*n* = 16) were collected from the first affiliated hospital of Soochow University. Histological assessment and grading of FL were determined by the proportion of centrocytes and centroblasts according to the WHO guidelines [11]. Different stages of FL consisted of grade 1 (*n* = 5), grade 2 (*n* = 8), grade 3A (*n* = 1), and grade 3B (*n* = 16). For immunohistological analysis, paraffin-embedded lymph node tissues from low-grade FL (n = 8) and high-grade FL (n = 8) were obtained from the secondary affiliated hospital of Soochow University. FL tissues included grade 1 (n = 1), grade 2 (*n* = 7), grade 3A (*n* = 3), and grade 3B (*n* = 5). Paired tissue samples (n = 5) were collected from FL patients transformed from a low-grade FL (grade 1 and grade 2) to a high-grade FL (grade 3A and 3B) or DLBCL. The project was approved by the ethics committee of Soochow University.

### Cell culture and transfection

Human FL-like cell line WSU-NHL was cultured in RPMI supplemented (HyClone, Logan city, Utah, USA) with 20% fetal bovine serum (FCS, Gibco Life Technologies, Carlsbad, CA, USA) and 1% penicillin/streptomycin (Corning, Discovery Boulevard Manassas, VA, USA) at 37 °C in a 5% CO2 atmosphere (Wugang, Shanghai, China). Mouse B cell lymphoma cell line A20 cells was cultured in RPMI supplemented with 20% FCS and 1% penicillin/streptomycin at 37 °C.

Mimic for miR-7e-5p (Supplemental Table 2, Guangzhou RiboBio, Guangzhou, China) were synthesized and transfected into both WSU-NHL and A20 cells using Lipofectamine® 3000 (Life Technologies) according to the manufacturer’s protocol with a media change 6 h after transfection. Small-interfering RNAs (siRNAs) for miR-7e-5p (Supplemental Table 2, Guangzhou RiboBio) were transfected by using Lipofectamine® 3000 (Life Technologies). Cells transfected with nonsense siRNA served as controls. RNAs and proteins were extracted 48 to 72 h post-transfect and used for further analysis.

Aclarubicin, which belongs to the anthracycline family and inhibits cell proliferation by inhibiting topoisomerase, is widely used for the treatment of B-cell lymphoma [12]. For the apoptosis assay, mice macrophage cells were treated with Aclarubicin (Shenzhen Main Luck Pharmaceuticals Inc. Shenzhen, China) at the concentration of 2.7 μg/ml for 2 h. Dimethysulphoxide (DMSO, Sigma-Aldrich, Saint Louis, Missouri, USA) treated cells was used as negative controls. Proteins were collected 48 h post-transfection to analyze the activation of caspase proteins. Cells were collected to determine the proportion of apoptotic cells by flow cytometry.

### Mouse experiment and macrophage isolation

The setup of mouse experiments was approved by the institutional regulation of Model Animal Research Center of Soochow University (Suzhou, China). Operation and termination of mice were performed according to the criteria of the animal welfare office of Soochow University.

Macrophages from mouse were isolated at the age of 6 weeks. Mice were injected with 1 ml liquid paraffin intraperitoneally 3 days before isolation according to Ray’s method [13]. At the time of isolation, mice were euthanized and sterilized with 70% ethanol. It was immediately followed by the intraperitoneally injection of 5 ml cold RPMI using a 27 g needle. After gently massage the peritoneum, ascitic fluid was collected using a 25 g needle. Peritoneum was opened by incision and the remaining fluid was collected by Pasteur pipette. Cell suspension was washed once with cold PBS and treated with RBC lysis buffer (Beyotime, Shanghai, China) for 1 min to get rid of erythrocytes. Three hours after seeding, the adherent cells were gently washed once with PBS and cultured in full RPMI medium for further analysis. Enriched macrophages were identified and counted by flow cytometry (CytoFLEX, Beckman Coulter, CA, USA).

### Real-time PCR and Western blotting

Isolation of miRNA from cultured cells was performed by using the HiPure Universal miRNA Kit (Magen biotechnology, Guangzhou, China). Total RNA was extracted from cells by 1 ml TRIZOL and miRNA were isolated according to the manufacturer’s instructions. To isolate miRNA from human tissues, 10 μm paraffin embedded tissues was deparaffinized by using xylene and followed by the isolation of miRNA using the MagMAX™ FFPE DNA/RNA Ultra Kit (Thermo Fisher Scientific, Woodward St, Austin). Proteins from cultured cells was extracted by using the 10x Cell lysis buffer (Cell Signaling Technology, MA, USA) and supplemented with 0.1 mM PMSF and proteinase-inhibitor (Cell Signaling Technology).

Reverse transcription for specific miRNAs was performed using 2 μg miRNA and respective primers for reverse transcription (listed in supplementary Table 2) according to the Stem-loop method [14]. Quantitative real-time PCR were carried out using the ABsolute qPCR SYBR Green ROX Mix (Thermo Fisher Scientific) and the following cycling condition: 95 °C for 10 min, 40 cycles of 95 °C for 15 s, and 60 °C for 60 s. StepOne Plus device (Thermo Fisher Scientific) was used for the detection. The small nuclearRNA U6 was used for normalization.

For Western blotting, 30 μg total proteins measured by BCA assay (Beyotime) was separated in sodium dodecyl sulfate (SDS) polyacrylamide gel electrophoresis and transferred to a PVDF membrane (Merck Millipore, MA, USA). It was followed by blocking the membrane with 5% skim milk in TBST (Tris-buffered saline/0.1% Tween 20). Membranes were incubated with primary antibody at 4 °C overnight and followed by the incubation of HRP-conjugated secondary antibody in skim milk (1:5000; Boster, Wuhan, China). Electronic chemical Laboratory (ECL) detection kit was used for the signal development (Merck Millipore).

### Chromatin Immunoprecipitation (ChIP)

After transfection, 1 × 10^7^ Cells in cultured T75 cell culture flask were collected and incubated with formaldehyde/PBS (1%) for 12 min to allow cross-linking of DNA and protein according to a ChIP protocol previously described [15]. Protein in the complex was diluted to 1 mg/ml with RIPA buffer and pre-cleared with 30 μl of Dynabeads Protein G (Nanoeast Biotech, Jiangsu, China). Another tube of 50 μl Dynabeads Protein G beads was blocked with 15 μg sperm DNA and 50 μg BSA. Pre-cleared samples were incubated with blocked Dynabeads and 4 μg primary antibody at 4 °C overnight to allow precipitation. The resulting immunocomplex-bound-beads were washed carefully with RIPA buffer and resuspended in TE buffer. Reversal crosslinking was achieved by incubation the complex in 4 M NaCl at 65 °C for 5 h. DNA was extracted and prepared for real-time PCRs analysis. Precipitated promoter fragments were normalized to a standard curve of genomic DNA. Primers binding to an unspecific sequence of genomic DNA was used as a negative control (listed in supplementary Table 2).

### Immunohistochemistry (IHC)

Immunohistochemical staining was carried out in paraffin embedded tissue from lymphoma patients. Tissue sections was deparaffinized and rehydrated by the following wash steps: 3 × 5 min xylene, 2 × 3 min 100% ethanol, 3 min 95% ethanol, 3 min 75% ethanol, and finally rinsing with aqua dest. 10 mM sodium citrate buffer (pH 6.0) (Boster, Wuhan, China) was incubated with tissue sections by microwave twice for 15 min. After retrieval of antigens, tissue sections were incubated with 30% peroxidase (Yonghua chemical technology, Jiangsu, China) for 10 min and blocked with 5% BSA-confining-liquid for 20 min (Boster) and incubated with primary antibody at 4 °C overnight. Tissue sections were incubated with a biotinylated anti-rabbit secondary antibody (Boster) for 2 h. Streptavidin-HRP (Boster) and Peroxidase Substrate (DAB) solution (MXB Biotechnologies, Fuzhou, China) were added to the tissue sections for signal development.

All IHC stains of lymphoma tissues were defined by two pathologist (H.N and LL.G) according to the intensity and percentage of positive tumor cells. Intensity was analyzed: 0 = negative, 1 = low nuclear stain, 2 = medium nuclear stain, 3 = strong nuclear stain. Percentage of positivity was defined: 0 = no positive cells, 1 = less than 1%, 2 = less than 10%, 3 = 10–50%, 4 = more than 50%. Final scoring was multiplying of qualitative and quantitative parameters.

### Flow Cytometry

For FACS analysis, macrophages in cell culture were detached with 0.25% trypsin (Gibco) to achieve a single-cell suspension in PBS solution. Followed by three times washing with PBS supplemented with 2% of FBS, cells were incubated with relevant antibodies or reagents. For the detection of different types of macrophages, isolated cell suspension was incubated with F4/80, CD11b, and CD86 antibodies for 30 min. After three times of washing with PBS, 1 × 10^5^ cells were resuspended in 100 μL PBS, and analyzed on flow cytometer (CytoFLEX, Beckman Coulter, CA, USA). Differentiated macrophages were identified by bright co-expression of F4/80 and CD11b. M1 macrophage was identified by the CD86^+^ population. Cell apoptosis analysis was performed using Annexin V-FITC Apoptosis Detection Kit (Beyotime), according to manufacturer’s instructions. For apoptosis analysis, 1 × 10^5^ cells were incubated with 5 μL fluorescein isothiocyanate (FITC)-labeled Annexin V and 10 μL propidium iodide (PI) for 20 min at room temperature (RT) in PBS containing 2% FBS. F4/80 was used as the marker for the gating of macrophages. Early apoptotic cells displayed a bright Annexin V and a dim PI, while late apoptotic cells had intensive stain of both reagents. Annexin V+/PI- events were acquired for the analysis of early apoptosis, while Annexin V+/PI+ events were used for the analysis of late apoptosis. Compensation control for negative population was achieved by incubation with each antibody/dye in separate vials. Data were analyzed using the CytExpert software (Ver. 2.3.0.84, CytExpert, Beckman Coulter, Inc).

### Data acquisition and statistical analysis

The data are presented as mean ± SD. Protein quantification after Western blotting was achieved by using the SkanIt™ Software (Thermo Fisher Scientific). Comparison of miRNA and proteins levels between non-paired groups were done by the nonparametric Mann-Whitney U test. Comparison of miRNA expression between paired groups of patients relied on the *t*-test. Significance levels were as following: *P* < 0.05, *P* < 0.01, and *P* < 0.001 (IBM SPSS Statistics 19.0, Armonk, NY, USA). Experiments were repeated three times for statistical analysis.

## Results

### miR-7e-5p is downregulated in FL progression to high-grade FL and DLBCL

**Fig. 1:**
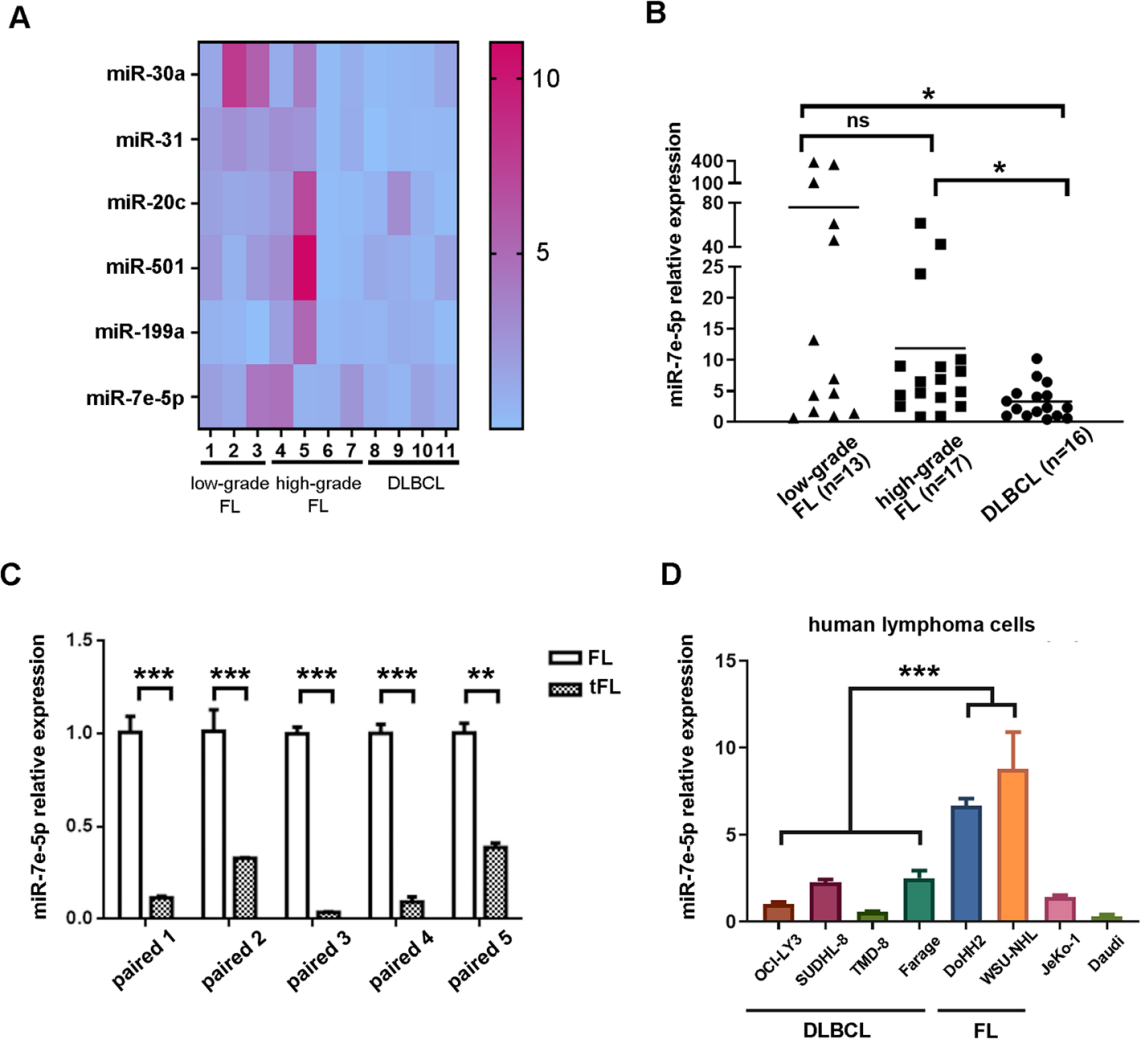
Downregulation of miR-7e-5p in the transformation of FL to DLBCL. **a** The expression of six miRNAs was compared in low-grade FL, high-grade FL and DLBCL tissue samples by real-time PCR. The median miRNA expression was calculated and is presented in the heatmap. Pink represents high expression, and blue represents low expression. **b** Differential expression of miR-7e-5p in low-grade FL, high-grade FL, DLBCL tissue samples by real-time PCR. Statistical test: Mann-Whitney U test. **c** Decreased expression of miR-7e-5p in five paired tissues with FL-tFL transformation. Statistical test: *t*-test. **d** The level of miR-7e-5p was decreased in FL cell lines compared to DLBCL cell lines. Statistical test: *t*-test

To identify miRNAs that may affect the transformation of indolent FL to high-grade FL and DLBCL, six miRNAs (miR-30a, miR-31, miR-20c, miR-501, miR-199a and miR-7e-5p) previously shown to be dramatically downregulated in FL transformation were selected as our candidates [10]. Tissue samples from different stages of FL (*n* = 7), which consisted of grade 1 (*n* = 2), grade 2 (*n* = 1), grade 3A (n = 1), and grade 3B (*n* = 3) were collected according to the WHO grading system [11]. Together with DLBCL, lymphoma tissues were divided into three groups, namely low-grade FL (grade 1–2, n = 3), high-grade FL (grade 3A and 3B, *n* = 4), and DLBCL (n = 4). All six of these miRNAs were downregulated in DLBCL compared with low-grade FL (Fig. 1a and Supporting Figure S1). Among the six miRNAs, miR-7e-5p showed trend of downregulation in both high-grade FL (fold change = 0.51, *P* = 0.077) and DLBCL (fold change = 0.39, *P* < 0.05) compared with low-grade FL, which suggests its role in the early stages of transformation. To validate the relevance of miR-7e-5p in the progression of lymphoma, we increased the number of samples from different stages (grade 1: *n* = 5, grade 2: *n* = 8, grade 3A: *n* = 1, grade 3B: *n* = 16, and DLBCL: n = 16), and miR-7e-5p was found to be significantly downregulated in DLBCL compared with low-grade FL (fold change = 0.04, *P* < 0.05), and compared with high-grade FL (fold change = 0.28, *P* < 0.05; Fig. 1b). However, the differential expression of miR-7e-5p was not detected between high-grade FL and low-grade FL. To avoid heterogeneity among patients, we further analyzed the expression of miR-7e-5p in five paired FL-tFL samples (Fig. 1c). In this cohort, miR-7e-5p was dramatically downregulated in the tFL samples compared with FL samples (fold change = 0.19, *P* < 0.001).

To further analyze the function of miR-7e-5p in FL progression in vitro, miRNA expression was compared in different lymphoma cell lines, namely, DLBCL cell lines (OCl-LY3, SUDHL-8, TMD-8, and Farage), FL cell lines (DoHH-2 and WSU-NHL), mantle cell lymphoma (JeKo-1) and Burkitt’s lymphoma (Daudi). As expected, the levels of miR-7e-5p were much higher in the FL cell lines than in the DLBCL cell lines (fold change = 4.89, *P* < 0.001; Fig. 1d). Taken together, these data revealed the potential role of miR-7e-5p in the suppression of the transformation of low-grade FL to high-grade FL and DLBCL.

### miR-7e-5p is negatively regulated by c-MYC during FL transformation

**Fig. 2:**
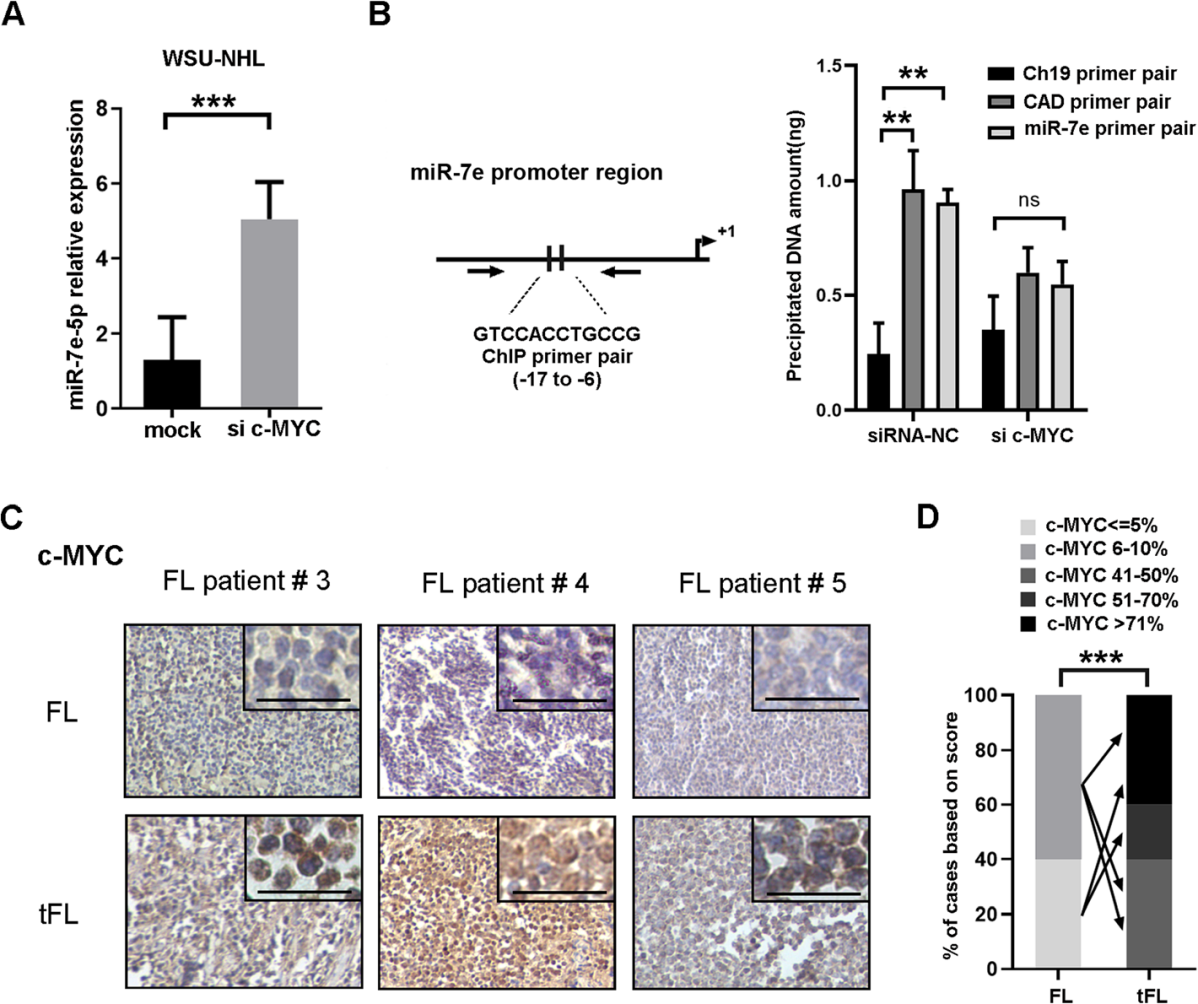
miR-7e-5p is transcriptionally repressed by c-MYC during FL transformation. **a** Higher levels of miR-7e-5p were detected in WSU-NHL cells after treatment with c-MYC siRNA (50 nM) for 36 h. Cells treated with transfection reagent only served as a negative control (Mock). Statistical test: Mann-Whitney U test. **b** Real-time PCR revealed the amount of DNA precipitated by anti-c-MYC antibody in WSU-NHL cells. The potential binding site of c-MYC and primers detecting the promoter region are indicated in the scheme. Ch: Chromosome, CAD: *Carbamoyl-Phosphate Synthetase-2*. Statistical test: Mann-Whitney U test. **c** Immunohistochemical staining of c-MYC in five paired tissues with FL-tFL transformation. Three representative tissues comparing the expression of c-MYC are shown. Scale bars: 100 μm. **d** Statistical analysis of c-MYC expression in five paired FL-tFL samples. The percentages of c-MYC-positive cells are shown. Each arrow indicates the change in c-MYC expression in paired samples with transformation. Statistical test: *t*-test

Genetic amplification of the oncogene c-MYC has been found to be a critical step of FL transformation via the induction of tumor growth and chromosomal instability [16]. Previously, c-MYC has been reported to regulate the expression of miR-7e-5p at the transcriptional level in cancer cell lines [17, 18]. We next analyzed the relevance of c-MYC in regulating miR-7e-5p expression in the progression of lymphoma. In WSU-NHL cells, quantitative PCR (q-PCR) revealed a 2.57-fold increase in miR-7e-5p expression after the inhibition of c-MYC by siRNA (*P* < 0.001; Fig. 2a and Supporting Figure S2). To test whether c-MYC regulates miR-7e-5p expression through direct interaction during transcription, a potential binding site of c-MYC in the promoter region of miR-7e (Fig. 2b; position − 17 to − 6) was predicted according to the JASPAR database [19]. Q-PCR of chromatin immunoprecipitation eluates revealed amplification of the promoter region of miR-7e compared to negative control (fold change = 3.69, *P* < 0.01), which was not detectable when c-MYC expression was inhibited by siRNA treatment. Primers detecting *Carbamoyl-Phosphate Synthetase-2* (CAD) served as the positive control for ChIP analysis. This indicates that miR-7e-5p is transcriptionally downregulated by c-MYC in WSU-NHL cells (Fig. 2b).

To confirm the relevance of c-MYC in the transformation of FL, c-MYC protein levels were detected in five paired samples from available patient tissues (Fig. 2c). Tumor tissues from tFL samples had strong c-MYC staining in the majority cells (> 40% cell positivity), while FL samples had weak or negative staining (0–10% cell positivity; Fig. 2c). Elevated expression of nuclear c-MYC was detected during FL transformation (*P* < 0.001; Fig. 2d). Taken together, these data demonstrate that miR-7e-5p is negatively regulated by c-MYC through direct binding to the miR-7e-5p promoter region during FL transformation.

### Exosomes mediate the transfer of miR-7e-5p from tumor cells to macrophages

**Fig. 3:**
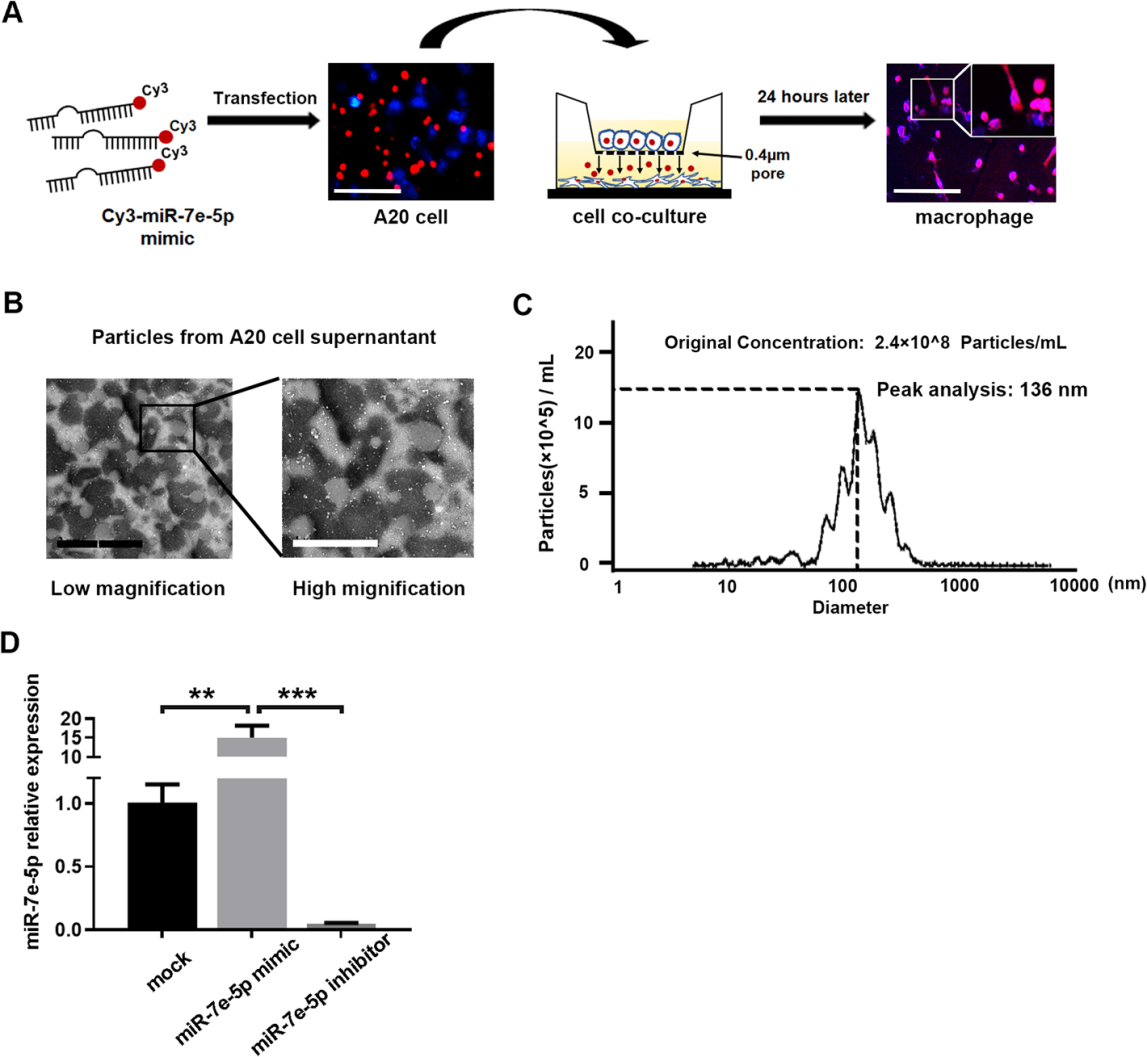
Transfer of miR-7e-5p from tumor cells to macrophages via exosomes. **a** Scheme showing the transfer of miR-7e-5p-mimics from A20 cells to mouse macrophages. A20 cells were transfected with 50 nM Cy3-miR-7e-5p-mimics (red fluorescence) and seeded in the upper chamber 6 h after transfection. Red fluorescence was detected in macrophages after coculturing with A20 cells for 24 h. Scale bars: 50 μm. **b** Images from transmission electron microscopy showed bilayer round-shaped particles with diameters ranging from 30 nm to 200 nm collected from the supernatant of A20 cells. Scale bars: 1 μm for lower magnification and 2 μm for higher magnification. **c** NanoSight particle tracking analysis (NTA) of exosomes revealed that most exosomes had a diameter of approximately 100 to 200 nm. The concentration of vesicles was 2.4 × 10^8^/ml at 150 nm. **d** Real-time PCR indicated the expression of miR-7e-5p in macrophages after incubation with exosomes extracted from A20 cells after 24 h. A20 cells were previously transfected with miR-7e-5p-mimics (50 nM) or inhibitors (100 nM) for 24 h. Cells treated with transfection reagent only served as a control (mock). Macrophages seeded on six-well plates were incubated with 1.2 × 10^7^ exosomes for 24 h. Statistical test: *t*-test

Remodeling of the tumor microenvironment has been shown to be an aspect as equally important as genetic modification in FL progression and is characterized by inactivation of cytotoxic T cells and macrophages and elevated levels of TFH cells [2, 3, 20]. We hypothesized that miR-7e-5p produced by tumor cells changes the immune context of FL, which may facilitate the survival of tumor cells. Previously, researchers found that lymphoma cells can modulate the phagocytic activity of classically activated macrophages (also known as M1 macrophages) [21]. To determine whether miR-7e-5p is responsible for the regulation of M1 macrophages, Cy3-labeled miR-7e-5p-mimics were transfected into the mouse lymphoma cell line A20, and the cells were then coincubated with mouse macrophages in a Transwell system with 0.4 μm pores. Flow cytometry analysis demonstrated that the majority (91.6%) of peritoneal macrophages were of the M1 phenotype (Supporting Figure S4). After 24 h of coincubation, obvious red fluorescence was detected in the cytoplasm of macrophages, demonstrating the transfer of miR-7e-5p-mimics into macrophages by lymphoma cell-derived particles (Fig. 3a).

Next, we evaluated whether exosomes were responsible for the transport of miR-7e-5p in the tumor microenvironment. Exosomes isolated from the supernatant of A20 cells were identified and visualized by both transmission electron microscopy (Fig. 3b) and NanoSight particle tracking analysis (Fig. 3c). After transfection of miR-7e-5p*-*mimics or inhibitors into A20 cells, exosomes carrying higher or lower levels of miR-7e-5p were collected and confirmed to express exosome markers (CD63 and CD81) by Western immunoblotting (Supporting Figure S6). Real-time PCR detected increased levels of mature miR-7e-5p in macrophages treated with exosomes carrying miR-7e-5p-mimics (fold change = 15, *P* < 0.01), whereas a reduced level of mature miR-7e-5p was detected in macrophages when its expression in lymphoma cells was inhibited by miR-7e-5p inhibitors (fold change = 0.05, *P* < 0.001; Fig. 3d and Supporting Figure S3). Taken together, these results demonstrate that miR-7e-5p was delivered from lymphoma cells to recipient macrophages.

### Exosomal miR-7e-5p inhibits FasL expression and inactivates the apoptotic pathway in stromal macrophages

**Fig. 4:**
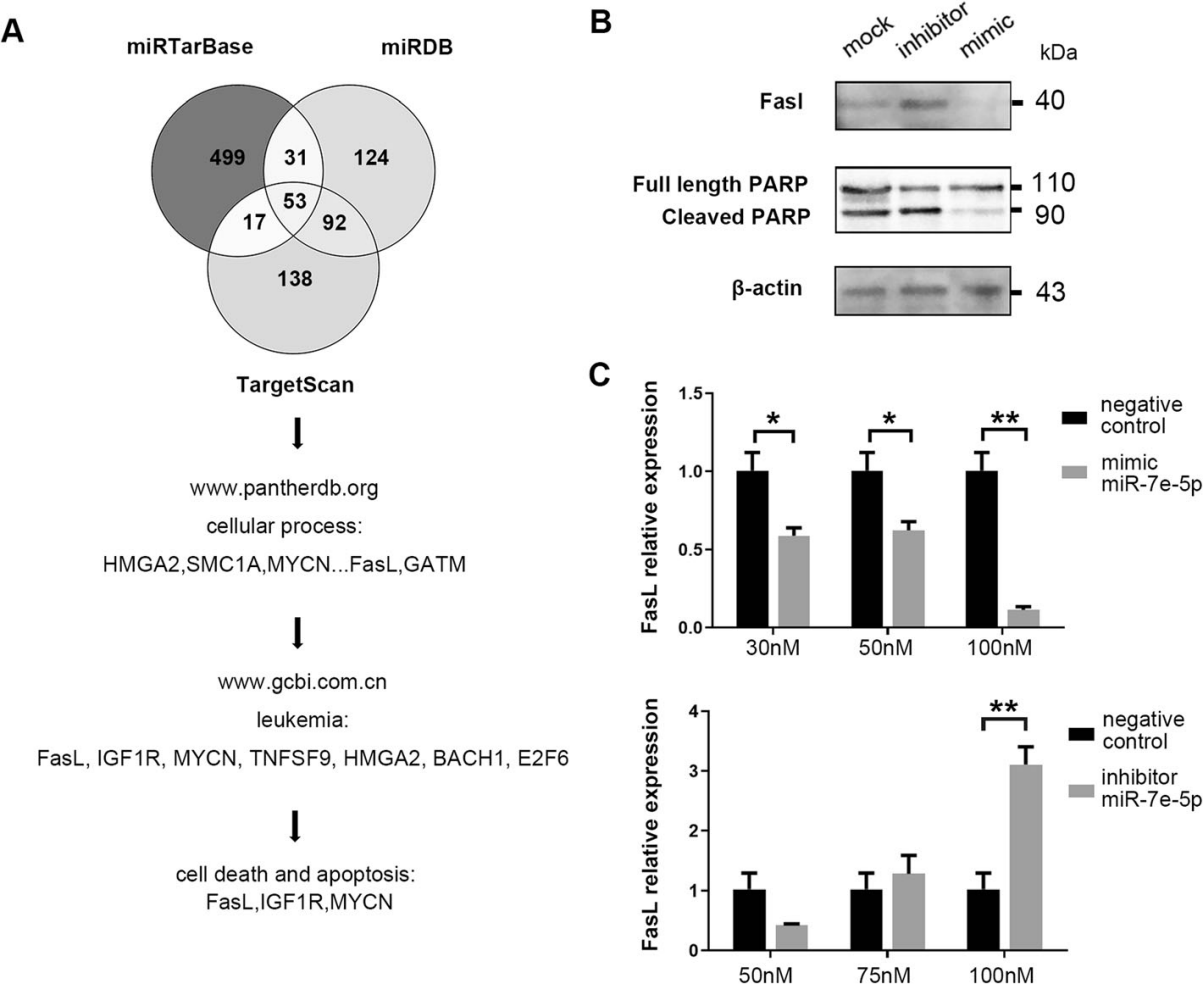
Exosomal miR-7e-5p inhibits macrophage apoptosis by targeting FASL. **a** Target genes of miR-7e-5p were predicted using miRTarBase, miRDB and TargetScan. Intersections of the results were used for functional analysis using the Panther and GCBI online databases. **b** Western blotting detecting PARP, cleaved PARP and FASL levels in macrophages is shown. The macrophages in each well were cocultured with miR-7e-5p-mimics or inhibitors-treated A20 cells. Actin served as a control. Macrophages were pretreated with aclarubicin at 2.67 μg/mL. **c** Real-time PCR analysis of FasL expression in macrophages. To each well of macrophages, 1.2 × 10^7^ exosomes from miR-7e-5p-mimics (30 nM, 50 nM, 100 nM) or inhibitors (50 nM, 75 nM, 100 nM)-treated A20 cells were added. Statistical test: *t*-test

To identify the target genes of miR-7e-5p, three miRNA target prediction databases (miRTarBase, TargetScan, and miRDB) were used for the identification of miR-7e-5p targets [22, 23]. Fifty-three potential target genes were found in all three databases, among which 21 had clear biological functions in cellular processes [24]. We further analyzed the relevance of the potential target genes in leukemia and lymphoma according to the GCBI database. Seven genes were associated with patient prognosis, among which *Fas ligand* (FasL), *N-myc proto-oncogene protein* (MYCN), *and insulin-like growth factor 1 receptor* (IGF1R) were associated with cell death and apoptosis (Fig. 4a).

FasL is a strong apoptotic factor that binds to its receptors to activate the caspase cascade and regulate the activation of proteins involved in cell proliferation (Holler et al., 2003). To evaluate the effect of miR-7e-5p on FasL expression, macrophages isolated from mice were coincubated with A20 cells previously transfected with miR-7e-5p-mimics or inhibitors. Western blotting detected a slight increase of FasL and weak cleavage of *poly-ADP-ribosyltransferase* (PARP) in macrophages when coincubated with inhibitors-treated A20 cells (Supporting Figure S5). With the presence of aclarubicin in cultured medium (2.7 μg/ml), Western blotting revealed obvious higher levels of FasL and cleaved PARP in the macrophages when miR-7e-5p production was inhibited in A20 cells, whereas decreased FasL and cleaved PARP were detected with miR-7e-5p-mimics treatment (Fig. 4b). We further evaluated the importance of exosomes in target gene regulation. Macrophages were incubated with exosomes isolated from A20 cells with differential expression of miR-7e-5p after miRNA-mimics or inhibitors treatments. Real-time PCR detected a downregulation of FasL in macrophages after incubation with exosomes from miR-7e-5p-mimic-expressing cells (fold change = 3.03, *P* < 0.01), whereas an upregulation of FasL was detected when macrophages were incubated with exosomes from miR-7e-5p inhibitors-treated cells (fold change = 0.11, *P* < 0.01; Fig. 4c). Taken together, these results suggest that miR-7e-5p targets FasL and suppresses the activity of the caspase cascade in M1 macrophages.

### Reduced exosomal miR-7e-5p induces apoptosis in stromal macrophages

**Fig. 5:**
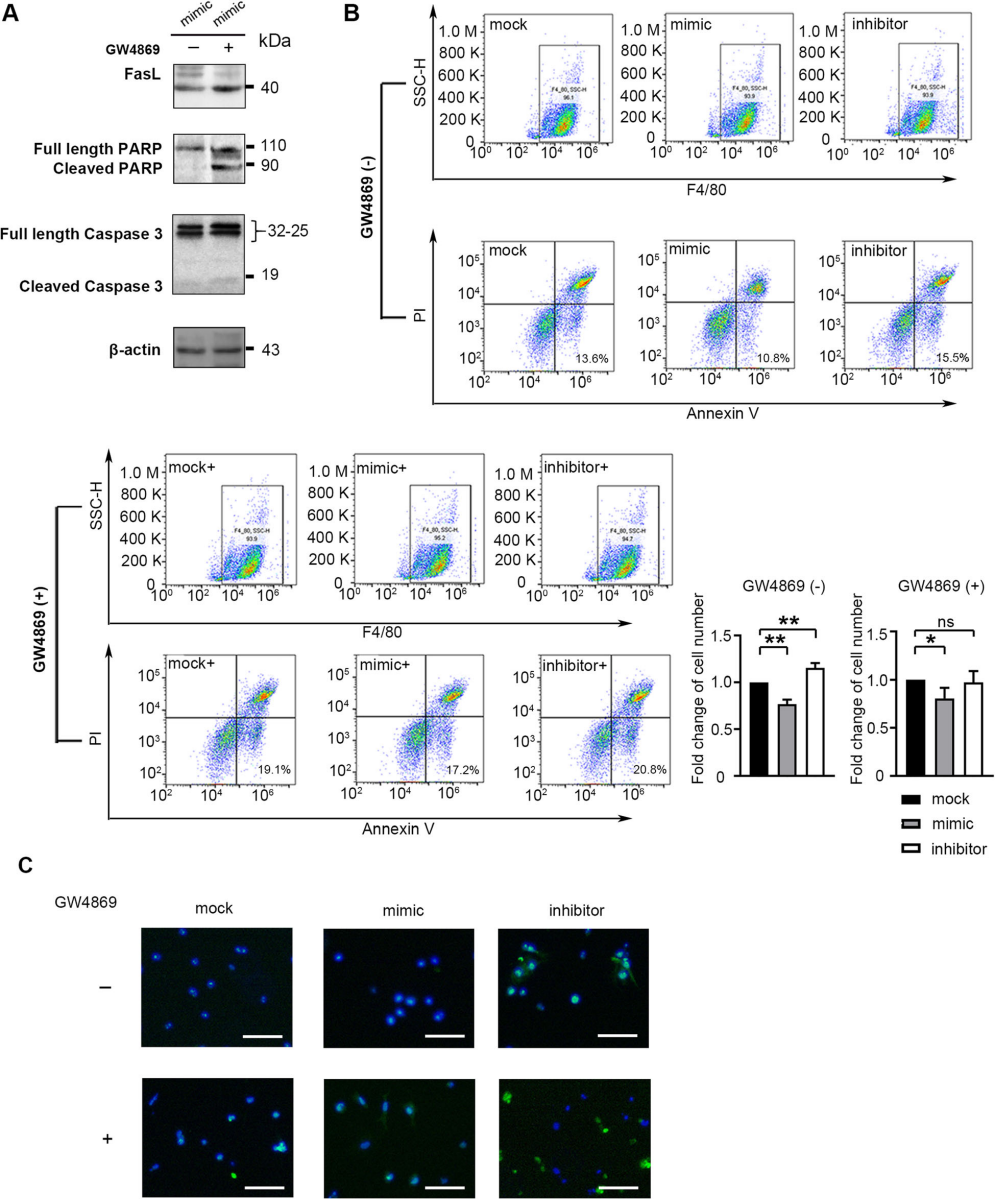
Treatment with exosomes influences the apoptosis of macrophages. **a** Western blotting detecting PARP, cleaved PARP, Caspase-3, cleaved Caspase-3 and FASL in macrophages treated with exosomes from A20 cells transfected with miR-7e-5p-mimics (50 nM). GW4869 (10 μM) was added to A20 cells for 24 h to inhibit exosome secretion. **b** Flow cytometry was used to detect apoptotic macrophages by FITC-Annexin V and propidium iodide (PI) double staining. F4/80 was used for the identification of macrophages. Bar charts indicating the change of cell populations undergoing early apoptosis according to three independent experiments. Statistic test: *t*-test. **c** Representative images of TUNEL assays to assess macrophage apoptosis. Macrophages were pretreated with aclarubicin at 2.67 μg/mL for 2 h before adding exosomes. Macrophages were incubated with terminal deoxynucleotidyl transferase (TdT) for 2 h before detection. Scale bars: 30 μm

Downregulation of exosomal miR-7e-5p led to the upregulation of FasL and activation of the apoptotic signaling pathway. To further confirm the effect of miR-7e-5p on cell apoptosis, macrophages were incubated with exosomes from differentially treated A20 cells in the presence of aclarubicin. Western blotting revealed a dramatic increase in FasL, cleaved PARP and Caspase 3 when exosomal miR-7e-5p was blocked by GW4869 treatment (Fig. 5a). Flow cytometry detected fewer early apoptotic macrophages in the group treated with exosomes from miR-7e-5p-mimics overexpressing cells compared with mock cells (fold change = 0.77, P < 0.01), while slight difference was detected when cells were treated with exosomes from miR-7e-5p-silenced cells (fold change = 1.15, P < 0.01; Fig. 5b). The effect of miR-7e-5p on the early apoptosis of macrophages was weakened in mimics overexpressing cells (fold change = 0.81, *P* < 0.05); and was abolished in miR-7e-5p-silenced cells when the release of exosomes was blocked by GW4869 (Fig. 5b). Similarly, in situ TUNEL assays detected less apoptosis in macrophages incubated with a high level of exosomal miR-7e-5p-mimics, while more apoptosis was detected when cells were incubated with exosomes carrying less miR-7e-5p via inhibitors treatment (Fig. 5c). The effect of miR-7e-5p on macrophages was restored when exosome production was blocked by GW4869 treatment (Fig. 5c). These results demonstrated that reduced exosomal miR-7e-5p levels promoted the apoptosis of recipient macrophages.

### miR-7e-5p and its target genes correlate with FL patient progression

**Fig. 6:**
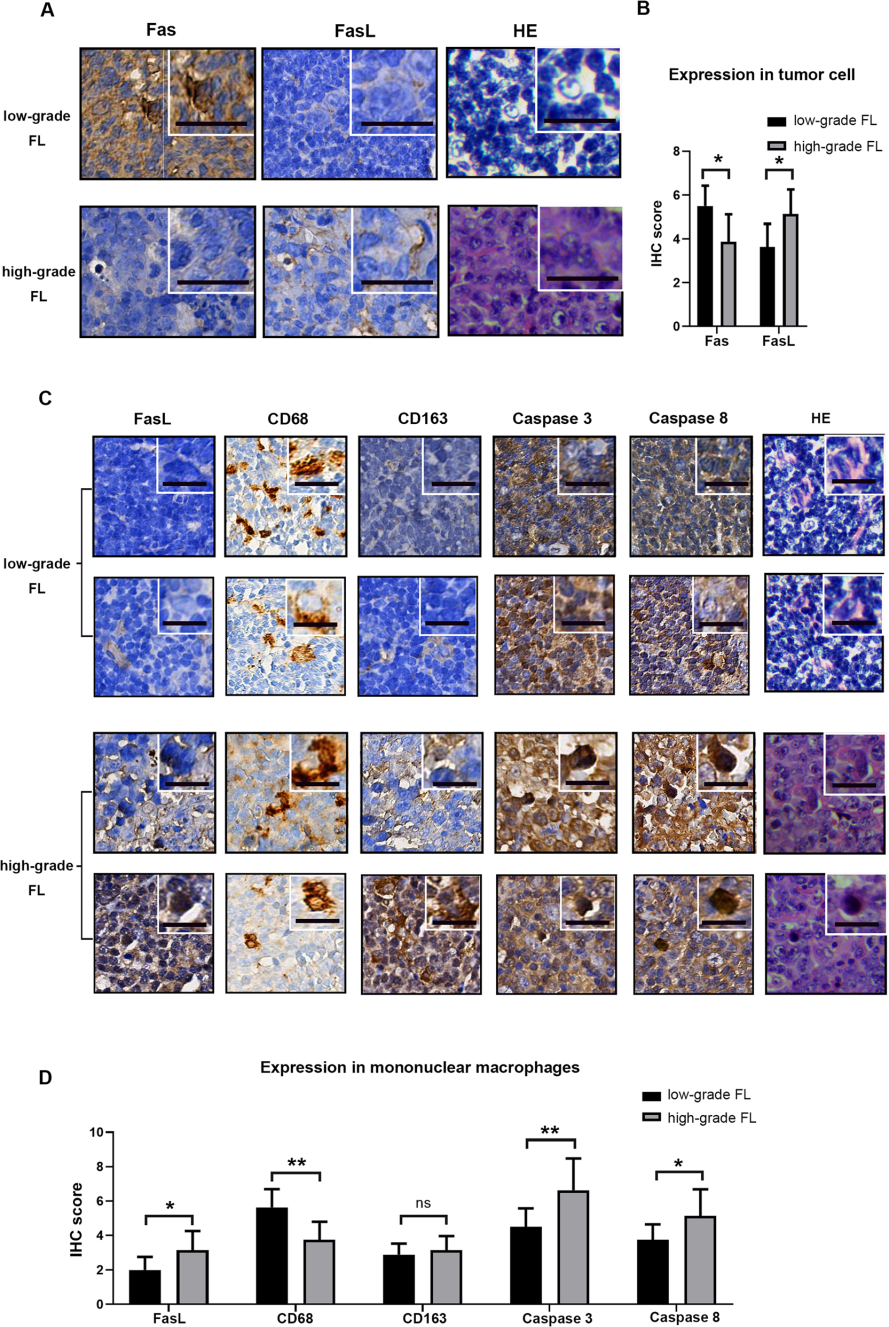
FasL increases the apoptosis of M1 cells by binding Fas in patients. **a** Representative example of HE and IHC stains for Fas and FasL expression in tumor cells in low-grade FL and high-grade FL patient tissues. Original magnification × 400, top right corner × 800. HE: hemotoxylin and eosin staining. Scale bars: 20 μm. **b** IHC score for Fas and FasL expression levels in tumor cells from eight low-grade FL samples and eight high-grade FL samples. Statistical test: *t*-test. **c** Representative example of HE and IHC stains for FasL, CD68, CD163, Caspase3 and Caspase 8 in macrophage cells in low-grade FL and high-grade FL patient tissues. Original magnification × 400, top right corner × 800. Scale bars: 20 μm. **d** IHC scores for FasL, CD68, CD163, Caspase3 and Caspase 8 expression levels in macrophage cells in eight low-grade FL and eight high-grade FL samples. Statistical test: *t*-test

Then, we tested whether the downregulation of miR-7e-5p and the upregulation of FasL were associated with the progression of FL. In the tumor areas, a loss of or weaker Fas expression and a clear extracellular FasL were detected in high-grade FL samples (Fig. 6a). A downregulation of Fas and upregulation of FasL were observed during the disease transformation (low-grade FL: *n* = 8, high-grade FL: n = 8, *P* < 0.05; Fig. 6b). In the tumor stroma, a decrease of M1 macrophages, an induction of M2 macrophages and an upregulation of miR-7e-5p downstream targets (FasL, Caspase 3 and Caspase 8) were detected in high-grade FL (Fig. 6c). The proportion of M1 macrophages was significantly lower; and the expression of miR-7e-5p target genes was higher in high-grade FL than in low-grade FL (low-grade FL: n = 8, high-grade FL: n = 8; Fig. 6d). Moreover, overexpression of miR-7e-5p was associated with unfavorable clinicopathological factors, including B symptoms and higher serum LDH levels (*n* = 46, *P* < 0.01; Table 1). These findings demonstrate that the decreased expression of miR-7e-5p is associated with the induction of FasL and apoptotic signaling in stromal immune cells, which leads to poorer clinical outcomes in FL patients.

**Table 1:** Correlation of miR-7e-5p expression. With clinicopathological variables in 46 cases

Variables	miR-7e-5p			P-value
	Total cases	Low expression(%)	High expression(%)	
Age (years)				
<=60	28	21 (56.8%)	7 (77.8%)	0.221
>60	18	16 (43.2%)	2 (22.2%)	
Sex				
Male	29	23 (62.2%)	6 (66.7%)	0.561
Female	17	14 (37.8%)	3 (33.3%)	
B symptom				
No	29	20 (54.1%)	9 (100.0%)	**0.009**
Yes	17	17 (45.9%)	0 (0%)	
Serum LDH				
Normal (<=225)	28	19 (51.4%)	9 (100.0%)	**0.006**
High (>225)	18	18 (48.7%)	0 (0.0%)	
Relapse or die in 5 years				
No	40	31 (83.8%)	9 (100.0%)	0.248
Yes	6	6 (16.2%)	0 (0.0%)	

*Abbreviations*: *LDH* lactic dehydrogenase. *P* values in bold are significant at *P*<0.05

## Discussion

The transformation of FL in 30% of patients to high-grade FL and DLBCL is widely accepted as an adverse event that dramatically reduces patient survival [1]. The decreased expression of different miRNAs, including miR-31, miR-7e, miR-30a and miR-199a, is associated with rapid disease progression, and these miRNAs are regarded as potential tumor suppressors in FL transformation [9, 10]. However, the underlying mechanisms by which miRNAs inhibit the malignant transformation of FL remain unclear. In this study, we found that miR-7e-5p is downregulated in lymphoma cells during the transformation of FL, which is responsible for the remodeling of the tumor microenvironment via the induction of immune escape and tumor progression.

The oncogene c-MYC is located at chromosome 8q24, which is amplified and translocated in 5 to 15% of DLBCL [25]. During the transformation of FL to high-grade FL and DLBCL, c-MYC is activated and consequently induces a subset of target genes involved in promoting cell growth and inhibiting apoptosis [16, 26]. Previously, let-7 family miRNAs were found to be widely repressed by c-MYC in B cell lymphoma [26]. The transcriptional regulation of miR-7e-5p by c-MYC was identified by an in vitro affinity purification approach in HEK293 cells [17]. In this study, we confirmed the direct binding of c-MYC to the miR-7e-5p promoter, which negatively regulated the transcription of miR-7e-5p and its tumor-suppressive activity in the transformation of FL. Therefore, the downregulation of miR-7e-5p during the transformation of FL to DLBCL can be explained by c-MYC amplification.

**Fig. 7:**
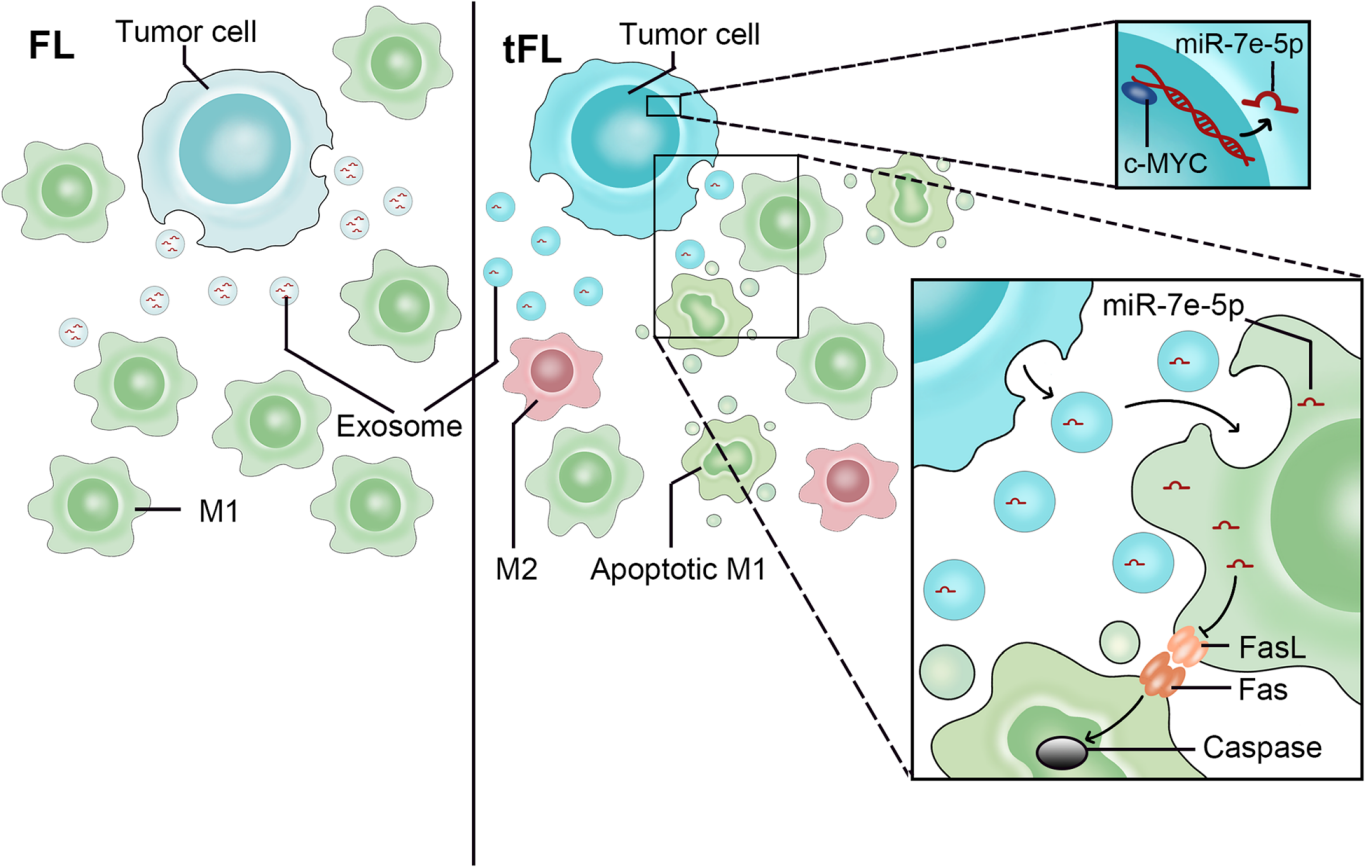
Downregulation of miR-7e-5p induces FL transformation via the activation of apoptotic signaling in macrophages. Scheme illustrating the mechanism the regulatory function of miR-7e-5p in recipient M1 macrophages. Downregulation of lymphoma-derived miR-7e-5p activates Fas-FasL interaction and downstream Caspase signaling in macrophages

We demonstrated the downregulation of miR-7e-5p in high-grade FL and DLBCL, which contributes to disease progression. The tumor suppressor role of miRNAs can be achieved by the direct inhibition of oncogenes in tumor cells and the regulation of the tumor cell microenvironment through exosome secretion [7, 8]. Our results reveal the importance of exosomes in mediating the transfer of miRNA from tumor cells to stromal cells. In the Transwell coculture system, miR-7e-5p was transferred from mouse lymphoma cells to macrophages. In this context, downregulation of miR-7e-5p in lymphoma cells led to a reduced amount of miR-7e-5p in surrounding macrophages. Therefore, miR-7e-5p expressed in normal tissue and indolent FL may function as a tumor suppressor; however, downregulation of miR-7e-5p in the transformation of lymphoma reduces the amount of miR-7e-5p and its function in recipient macrophages (Fig. 7).

Based on miRNA databases and enrichment analysis, FasL was selected as a potential target gene of miR-7e-5p. FasL belongs to the tumor necrosis factor (TNF) family, which interacts with and activates Fas receptor (Fas) and induces the caspase cascade during cellular apoptosis [27]. By binding to its ligand, the intracellular *death domain* of Fas interacts with each other and serves as a platform for the initiation of apoptosis through the enrichment of Caspase-8 protein [28]. After dimerization, Caspase-8 undergoes autocatalytic cleavage and triggers the activation of Caspase-3 and Caspase-7, which affects the function of many genes (e.g., PARP) involved in cell proliferation and transformation [28]. As expected, we detected an inhibition of FasL expression at both the transcriptional and protein levels in recipient macrophages with elevated exosomal miR-7e-5p levels in the stroma. Consequently, the cleavage of PARP was reduced in the coincubation experimental setup with miR-7e-5p-mimic overexpression. Interestingly, the reduction in cleaved PARP and cleaved Caspase-3 was more obvious in macrophages treated with isolated exomes containing a high level of miR-7e-5p. The negative regulation of macrophage apoptosis by exosomal miR-7e-5p was demonstrated by FACS analysis and in situ TUNEL assays. Overall, sufficient exosomal miR-7e-5p facilitates the survival of macrophages. The decreased production of exosomal miR-7e-5p in lymphoma cells activates apoptotic signaling via its target FasL in stromal macrophages (Fig. 7).

Although FasL is upregulated by the suppressive effect of miR-7e-5p during FL transformation, it probably cannot activate the apoptosis of lymphoma cells due to multiple immune escape mechanisms [2]. Loss of Fas is present in 17% of FL and 51% of DLBCL, while the *death domain* of FAS is mutated in 6% of FL and 20% of DLBCL, which leads to inactivation of the Fas-mediated apoptotic signaling pathway [29]. However, upregulation of FasL in macrophages according to our results induces immune suppression due to the Fas-FasL interaction. In addition, FasL was previously found to bind to and form a hexamer with *cytotoxic T lymphocyte associated protein 4* (CTLA4), which abrogated costimulation by binding to B7 receptor-expressing immune cells [30]. Thus, FasL induces a strong apoptotic signal not only by activating the caspase cascade but also by switching off antiapoptotic signaling in immune cells [30]. Our clinical data demonstrated that repression of miR-7e-5p was associated with the upregulation of FasL and apoptotic signaling in M1 macrophages, accounting for poorer clinical outcomes.

In FL and DLBCL, the translocation and activation of c-MYC and *B-cell lymphoma 2* (BCl2) is regarded as “double-hit lymphoma” because of the poor survival of patients with this pattern of genetic alteration [31]. In FL, t (14; 18) was reported in 96% of patients, which was associated with activation of BCl2 and apoptosis inhibition [29]. Our results demonstrate that the activation of c-MYC in lymphoma cells drives immune suppression through the downregulation of exosomal miRNA-7e-5p and activation of FAS-mediated apoptosis in macrophages. In this manner, the oncogene c-MYC not only promotes tumor cell proliferation but also collaborates with the antiapoptotic factor BCl2 in escaping immune surveillance by nearby immune cells. For the treatment of FL, aclarubicin inhibits DNA replication in cells and is currently used as a standard chemotherapy for patients [32]. The effects of FasL expression and cell apoptosis in macrophages were performed under aclarubicin treatment to mimic the situation in the clinic, which revealed the importance of considering the immune microenvironment when planning appropriate treatment for FL patients.

## Conclusion

In summary, our study revealed the downregulation of miR-7e-5p during the transformation of FL to aggressive lymphoma. In tumor microenvironment, miR-7e-5p was transferred from lymphoma cells to stromal macrophage via exosome. In stromal M1 macrophages, reduced miR-7e-5p level led to the upregulation of FasL, the activation of caspase signaling, and the cleavage of PARP protein, which promoted macrophage apoptosis. In patient tissues, upregulation of FasL and activation of caspase signaling was detected in tumor stromal macrophages. The downregulation of miR-7e-5p correlated with poorer clinical outcomes. These findings indicated potential therapeutic function of miR-7e-5p in restoring macrophage surveillance against FL progression.

## Supplementary Information

**Additional file 1 : Supporting Figure S1.** Relative miRNA expression in low-grade FL, high-grade FL and DLBCL. Statistical test: Mann-Whitney U test.

**Additional file 2 : Supporting Figure S2.** The expression level of c-MYC after transfecting three different siRNA sequences. **A)** Western blotting indicating the protein levels of c-MYC after siRNA inhibition. **B)** Real-time PCR indicating the mRNA levels of c-MYC after siRNA treatment. Statistical test: *t*-test.

**Additional file 3 : Supporting Figure S3.** Verification of efficiency of miR-7e-5p-mimics and inhibitors treatment. Left: The level of miR-7e-5p expression increased significantly after treatment of miR-7e-5p-mimics. Right: Lower expression level of miR-7e-5p was detected after transfection of the miR-7e-5p inhibitors. Statistical test: *t*-test.

**Additional file 4 : Supporting Figure S4.** FACS analysis of cell types isolated from mouse abdominal. Left: population of cells with F4/80 and CD11b co-expression (41%). Right: histogram of CD86+ events indicating the population of M1 macrophages.

**Additional file 5 : Supporting Figure S5.** Effect of miR-7e-5p expression of A20 cells on apoptotic signaling in macrophages. Western blotting detecting FASL, PARP, cleaved PARP in macrophages. The macrophages were cocultured with miR-7e-5p-mimics or inhibitors-treated A20 cells. Macrophages were pretreated with DMSO as the negative control for aclarubicin.

**Additional file 6 : Supporting Figure S6.** Expression of CD81 and CD63 in exosomes from A20 cells. CD81 (30 kDa) and CD63 (35 kDa) protein levels in the exosomes from A20 cells, which were treated with miR-7e-5p-mimics or inhibitors. Transferrin (80 kDa) severed as a loading control. GW4869 was used to inhibit exosome secretion from A20 cells.

**Additional file 7: Supplementary Table S1**. Antibodies.

**Additional file 8 : Supplementary Table S2**. Primers and siRNAs

## Data Availability

Additional Supporting Information are provided in Supporting Figure 1, 2, 3, 4, 5, 6. Antibodies, sequence for miRNA mimics, inhibitors and primers are listed in Supporting Table S1–S2.

## Abbreviations

FL: Follicular lymphoma
tFL: transformed follicular lymphoma
DLBCL: Diffuse large B-cell lymphoma
FasL: Fas ligand
PARP: Poly-ADP-ribosyltransferase
