# Supplementary figures and images for: MiR-7e-5p downregulation promotes transformation of low-grade follicular lymphoma to aggressive lymphoma by modulating an immunosuppressive stroma through the upregulation of FasL in M1 macrophages
Source: J Exp Clin Cancer Res. 2020 Nov 9;39:237. doi: 10.1186/s13046-020-01747-z (PMC7654609; doi:10.1186/s13046-020-01747-z)

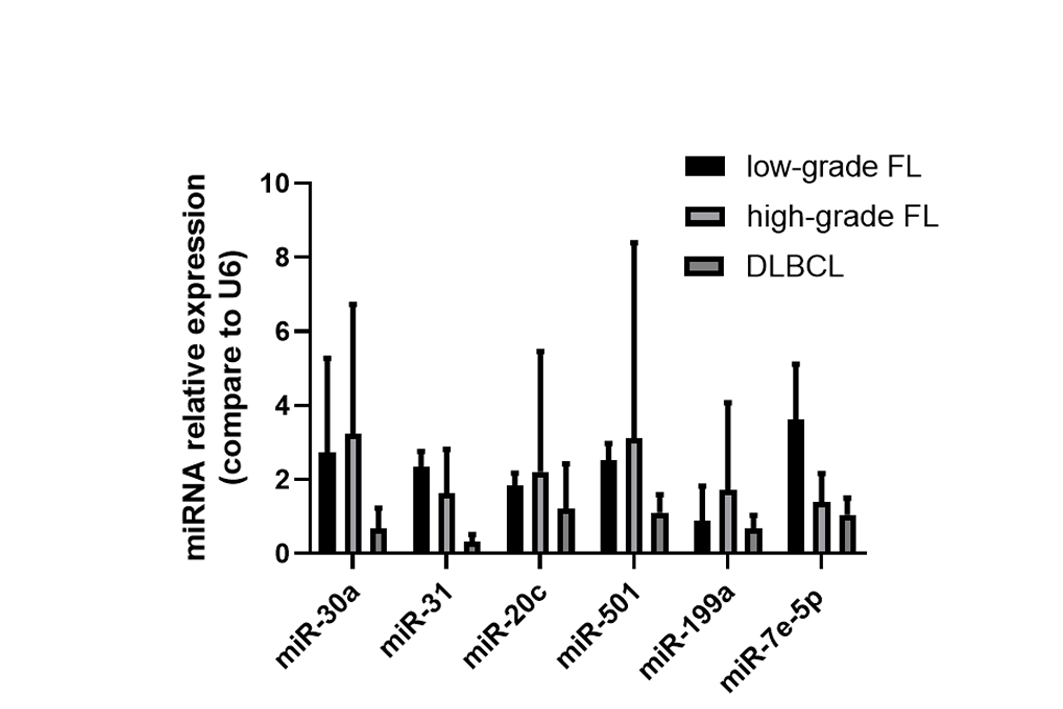

Supplement: Supplementary file 1 — Additional file 1 : Supporting Figure S1. Relative miRNA expression in low-grade FL, high-grade FL and DLBCL. Statistical test: Mann-Whitney U test. [file 13046_2020_1747_MOESM1_ESM.tif]

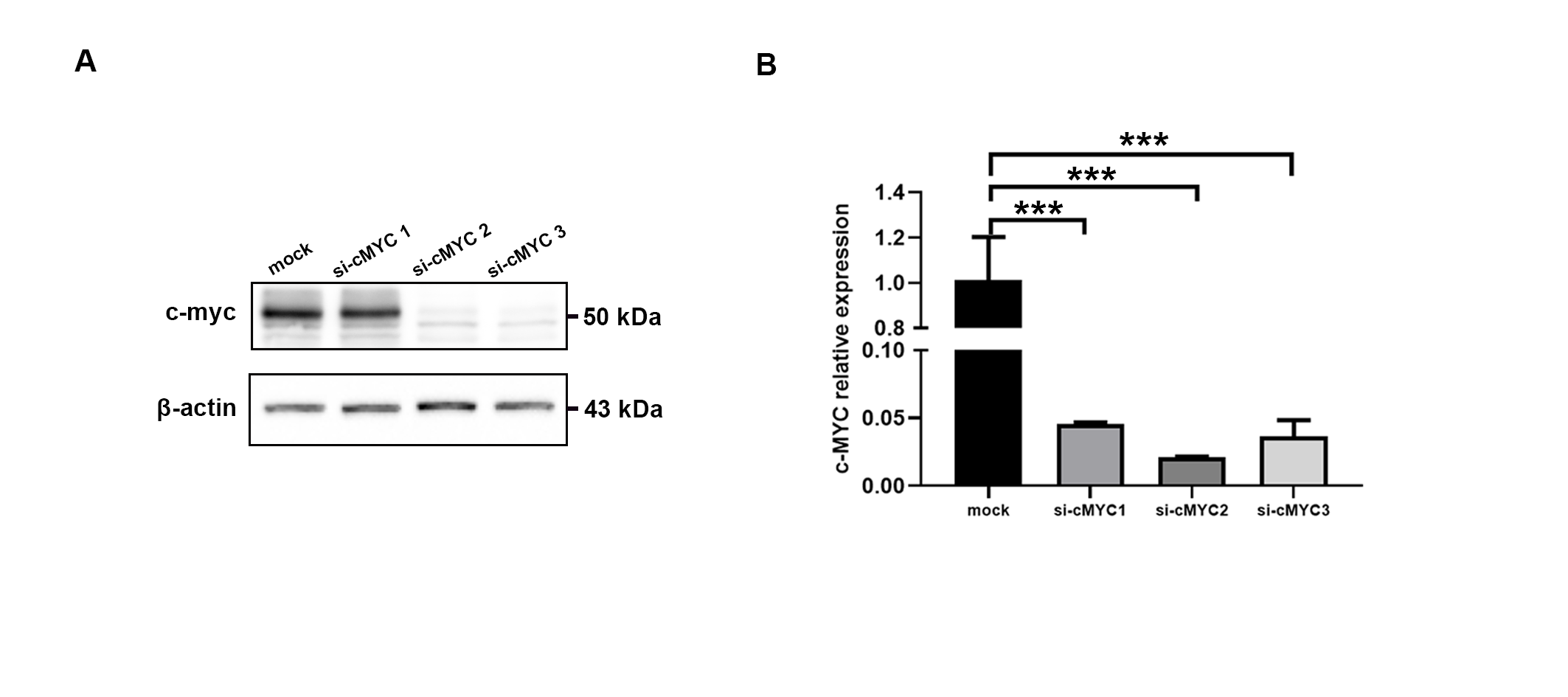

Supplement: Supplementary file 2 — Additional file 2 : Supporting Figure S2. The expression level of c-MYC after transfecting three different siRNA sequences. A) Western blotting indicating the protein levels of c-MYC after siRNA inhibition. B) Real-time PCR indicating the mRNA levels of c-MYC after siRNA treatment. Statistical test: t-test. [file 13046_2020_1747_MOESM2_ESM.tif]

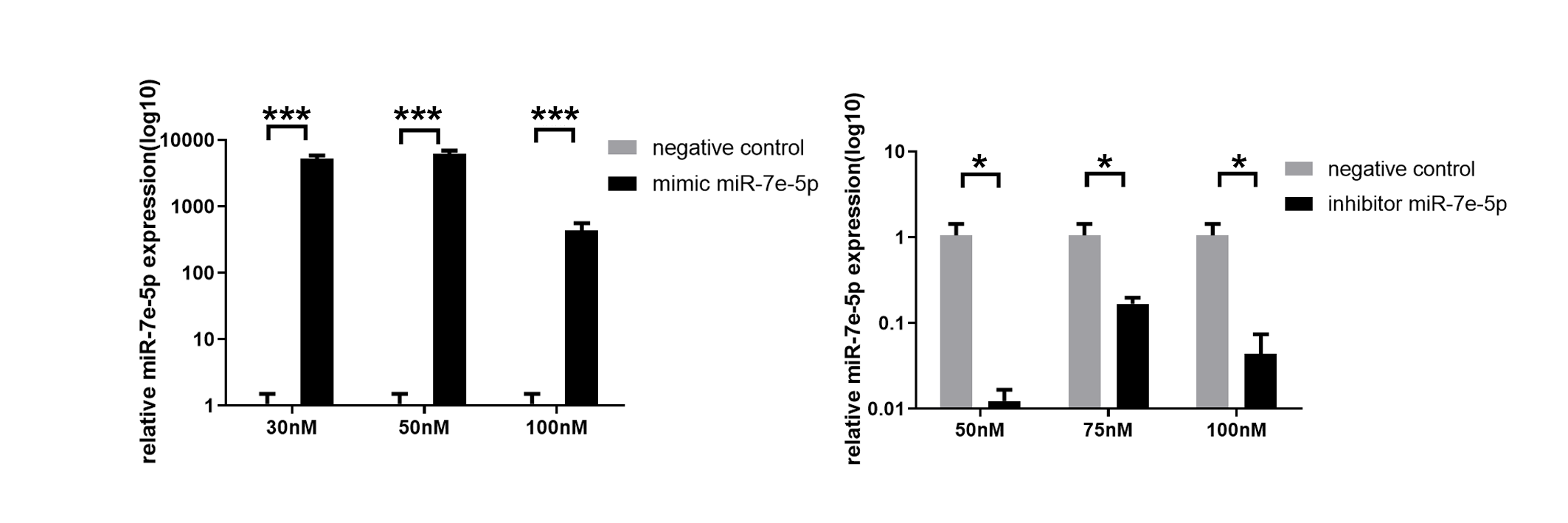

Supplement: Supplementary file 3 — Additional file 3 : Supporting Figure S3. Verification of efficiency of miR-7e-5p-mimics and inhibitors treatment. Left: The level of miR-7e-5p expression increased significantly after treatment of miR-7e-5p-mimics. Right: Lower expression level of miR-7e-5p was detected after transfection of the miR-7e-5p inhibitors. Statistical test: t-test. [file 13046_2020_1747_MOESM3_ESM.tif]

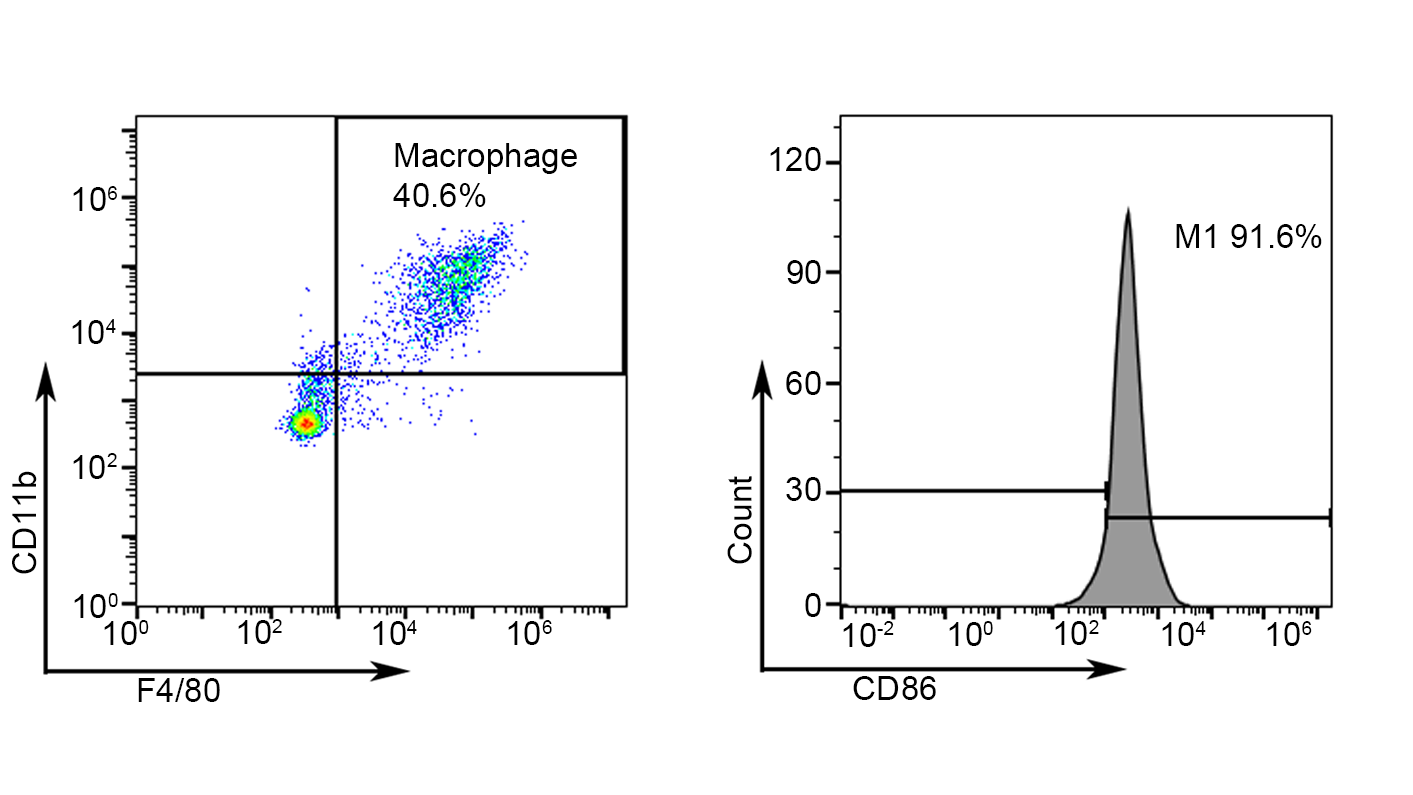

Supplement: Supplementary file 4 — Additional file 4 : Supporting Figure S4. FACS analysis of cell types isolated from mouse abdominal. Left: population of cells with F4/80 and CD11b co-expression (41%). Right: histogram of CD86+ events indicating the population of M1 macrophages. [file 13046_2020_1747_MOESM4_ESM.tif]

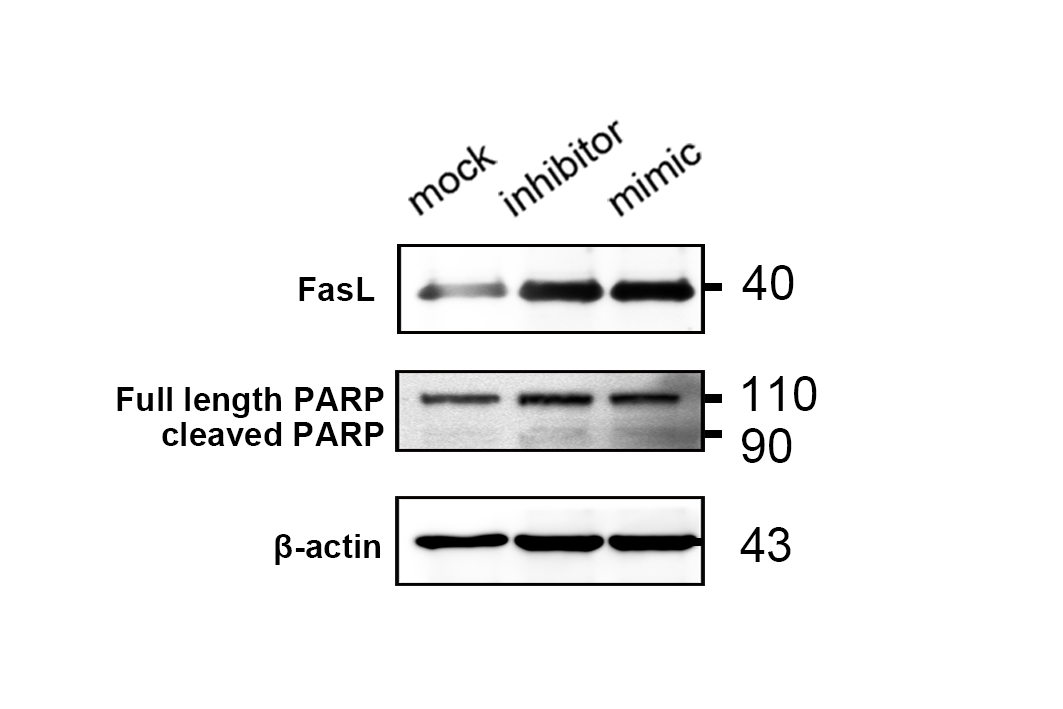

Supplement: Supplementary file 5 — Additional file 5 : Supporting Figure S5. Effect of miR-7e-5p expression of A20 cells on apoptotic signaling in macrophages. Western blotting detecting FASL, PARP, cleaved PARP in macrophages. The macrophages were cocultured with miR-7e-5p-mimics or inhibitors-treated A20 cells. Macrophages were pretreated with DMSO as the negative control for aclarubicin. [file 13046_2020_1747_MOESM5_ESM.tif]

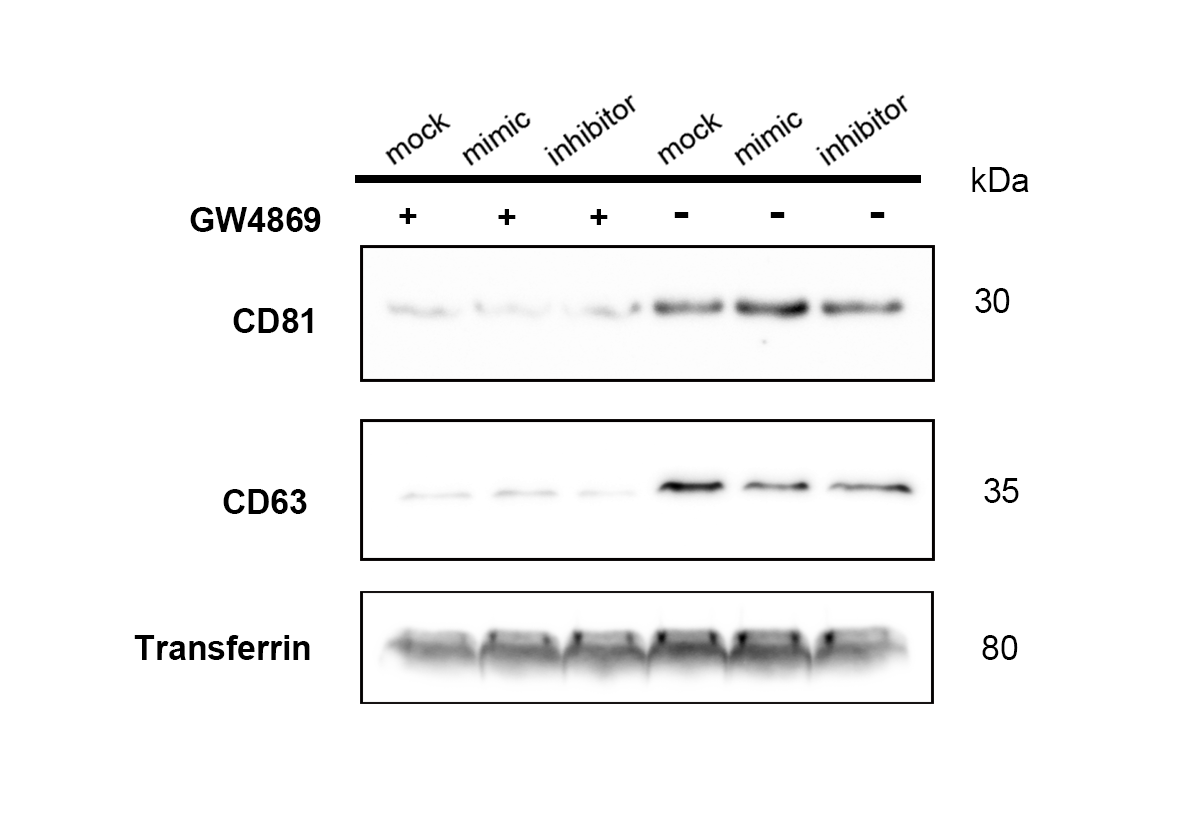

Supplement: Supplementary file 6 — Additional file 6 : Supporting Figure S6. Expression of CD81 and CD63 in exosomes from A20 cells. CD81 (30 kDa) and CD63 (35 kDa) protein levels in the exosomes from A20 cells, which were treated with miR-7e-5p-mimics or inhibitors. Transferrin (80 kDa) severed as a loading control. GW4869 was used to inhibit exosome secretion from A20 cells. [file 13046_2020_1747_MOESM6_ESM.tif]
