# Supplementary material for: MiR-7e-5p downregulation promotes transformation of low-grade follicular lymphoma to aggressive lymphoma by modulating an immunosuppressive stroma through the upregulation of FasL in M1 macrophages
Source: J Exp Clin Cancer Res. 2020 Nov 9;39:237. doi: 10.1186/s13046-020-01747-z (PMC7654609; doi:10.1186/s13046-020-01747-z)
Supplement: Supplementary file 7 — Additional file 7: Supplementary Table S1. Antibodies. [file 13046_2020_1747_MOESM7_ESM.docx]

**Supplementary Table S1: Antibodies**

| **Antigen (clone)** | **Company** | **Concentration/Amount** | **Application** |
| --- | --- | --- | --- |
| c-MYC (10828-1-AP) | Proteintech | 0.5 µg/ml | IHC, ChIP |
| CD81([sc-166029](https://www.scbt.com/zh/p/cd81-antibody-b-11?requestFrom=search)) | Santa Cruz Biotechnology | 3 µg | ChIP |
| CD63(25682-1-AP) | Proteintech | 0.5 µg/ml | IHC |
| FasL([PA1576](http://www.boster.com.cn/product/anti-faslg-antibody_pa1576.html)) | [BOSTER Biological Technology](http://www.baidu.com/link?url=74kXHDCzXDG0BaLv3Etq_itQjYTbbUWGSRBus6LV-D8IXUwkPQuDS5K91MtV-mXa) | 3 µg | WB |
| FasL(bs-0216R) | BIoss Antibody | 5 µg/ml | IHC |
| Fas(60196-1-lg) | Proteintech | 0.5 µg/ml | IHC |
| PARP([9532](https://www.cst-c.com.cn/products/primary-antibodies/parp-46d11-rabbit-mab/9532?site-search-type=Products)) | Cell Signaling | 3 µg | WB |
| Caspase 3(19677-1-AP) | Proteintech | 0.5 µg/ml | WB, IHC |
| Caspase 8 (13423-1-AP) | Proteintech | 0.5 µg/ml | IHC |
| CD163(16646-1-AP) | Proteintech | 0.5 µg/ml | WB |
| β-actin (BA2305) | [BOSTER Biological Technology](http://www.baidu.com/link?url=74kXHDCzXDG0BaLv3Etq_itQjYTbbUWGSRBus6LV-D8IXUwkPQuDS5K91MtV-mXa) | 3 µg | WB |
| HRP Conjugated AffiniPure Goat Anti-rabbit/mouse IgG (H+L) (BA1056) | [BOSTER Biological Technology](http://www.baidu.com/link?url=74kXHDCzXDG0BaLv3Etq_itQjYTbbUWGSRBus6LV-D8IXUwkPQuDS5K91MtV-mXa) | 1 µg/ml | WB |
| [Biotin Conjugated AffiniPure Goat Anti-Mouse IgG(H+L)](http://www.boster.com.cn/product/biotin-conjugated-affinipure-goat-anti-mouse-igg-h-l_ba1001.html)(BA1001) | [BOSTER Biological Technology](http://www.baidu.com/link?url=74kXHDCzXDG0BaLv3Etq_itQjYTbbUWGSRBus6LV-D8IXUwkPQuDS5K91MtV-mXa) | 10 µg/ml | IHC |
| HRP Conjugated Streptavidin (BA1088) | [BOSTER Biological Technology](http://www.baidu.com/link?url=74kXHDCzXDG0BaLv3Etq_itQjYTbbUWGSRBus6LV-D8IXUwkPQuDS5K91MtV-mXa) | 10 µg/ml | IHC |
| Brilliant Violet 421™ anti-mouse/human CD11b Antibody (101251) | Biolegend | 0.25 µg | FC |
| APC anti-mouse F4/80 Antibody (123115) | Biolegend | 0.25 µg | FC |
| FITC anti-mouse CD86 Antibody (105005) | Biolegend | 1.0 µg | FC |
| PE anti-mouse CD163 Antibody (155307) | Biolegend | 0.25 µg | FC |

WB: Western Blotting, ChIP: Chromatin Immunoprecipitation, IHC: Immunohistochemistry, FC: Flow Cytometry
