# Supplementary material for: MiR-7e-5p downregulation promotes transformation of low-grade follicular lymphoma to aggressive lymphoma by modulating an immunosuppressive stroma through the upregulation of FasL in M1 macrophages
Source: J Exp Clin Cancer Res. 2020 Nov 9;39:237. doi: 10.1186/s13046-020-01747-z (PMC7654609; doi:10.1186/s13046-020-01747-z)
Supplement: Supplementary file 8 — Additional file 8 : Supplementary Table S2. Primers and siRNAs [file 13046_2020_1747_MOESM8_ESM.docx]

| **Real-time PCR Primers** | | | |
| --- | --- | --- | --- |
| **Gene** | **Accession Number** | **Sequence forward primer (5´-3´)** | **Sequence reverse primer (5´-3´)** |
| hsa-miR-501-3P |  | GAAACCTTTGTCCCTGGGTGAGA | GTGCGTGTCGTGGAGTCG |
| hsa-miR-199a-5P |  | GAAACCAGTGTTCAGACTACCTGTTC | GTGCGTGTCGTGGAGTCG |
| hsa-miR-204-5P |  | GGAATTCCCTTTGTCATCCTATGCCT | GTGCGTGTCGTGGAGTCG |
| hsa-miR-30a-5p |  | GGAAGACATAGTTGCAAGATGGG | GTGCGTGTCGTGGAGTCG |
| hsa-miR-31 |  | GGAAGGCAAGATGCTGGCATAGCT | GTGCGTGTCGTGGAGTCG |
| hsa-miR-7e-5p |  | GGAATGAGGTAGGAGGTTGTATAGTT | GTGCGTGTCGTGGAGTCG |
| c-MYC | NM_032133 | GGATTCCCGCCTCAGAATAAC | GTGGGTGTGGGTTGTTCAGG |
| FasL | NM_000639 | TGCCTTGGTAGGATTGGGC | GCTGGTAGACTCTCGGAGTTC |

**Supplementary Table S2: Primers and siRNAs`**

**Stem loop primers for miRNA reverse transcription**

| **Gene** | **Primer Sequence (5‘-3‘)** |
| --- | --- |
| hsa-miR-501-3P | GTCGTATCCAGTGCGTGTCGTGGAGTCGGCAATTGCACTGGATACGACTCTCAC |
| hsa-miR-199a-5P | GTCGTATCCAGTGCGTGTCGTGGAGTCGGCAATTGCACTGGATACGACGAACAG |
| hsa-miR-204-5P | GTCGTATCCAGTGCGTGTCGTGGAGTCGGCAATTGCACTGGATACGACAGGCAT |
| hsa-miR-30a-5p | GTCGTATCCAGTGCGTGTCGTGGAGTCGGCAATTGCACTGGATACGACCCCATC |
| hsa-miR-31 | GTCGTATCCAGTGCGTGTCGTGGAGTCGGCAATTGCACTGGATACGACAGCTAT |
| hsa-miR-7e-5p | GTCGTATCCAGTGCGTGTCGTGGAGTCGGCAATTGCACTGGATACGACAACTAT |

| **PCR primers for ChIP** | |
| --- | --- |
| **Name** | **Sequence (5´-3´)** |
| hsa-miR-7e forward | TCCCTGTCTGTCTGTCTGTC |
| hsa-miR-7e reverse | TCCTCAACTATACAACCTCCTAC |
| CAD forward | GTGGTTCCAGTGGAGTTTGCAGT |
| CAD reverse | GGATAAGGTCTGCAGCCTAAC |
| Chromosome 19 forward | CTCTGCTTCACAACCTACTCTGAG |
| Chromosome 19 reverse | GATGAAGTGTGGATGGTCTCCT |

| **Sequence of siRNAs** | | |
| --- | --- | --- |
| **Gene name** | **Accession number** | **Target sequence** |
| c-MYC #1 | NM_032133 | GAGGAGACATGGTGAACCA |
| c-MYC #2 | NM_032133 | GGGTCAAGTTGGACAGTGT |
| c-MYC #3 | NM_032133 | CGACGAGACCTTCATCAAA |
|  |  |  |

| **Sequence of miRNA mimics and inhibitors** | | |
| --- | --- | --- |
| **Gene name** | **Sense (5´- 3´)** | **Antisense (5´- 3´)** |
| hsa-let-7e-5p mimics | UGAGGUAGGAGGUUGUAUAGUU | AACUAUACAACCUCCUACCUC |
| hsa-let-7e-5p inhibitors |  | AACUAUACAACCUCCUACCUCA |
